# Promoting Health in a Rural Community in the Basque Country by Leveraging Health Assets Identified through a Community Health Diagnosis

**DOI:** 10.3390/ijerph19020627

**Published:** 2022-01-06

**Authors:** Maria Jose Alberdi-Erice, Esperanza Rayón-Valpuesta, Homero Martinez

**Affiliations:** 1 Facultad de Medicina y Enfermería, Sección Donostia, Universidad Pública del País Vasco (UPV), 20014 San Sebastián, Spain; mariajose.alberdi@ehu.eus; 2 Facultad de Enfermería, Fisioterapia y Podología, Universidad Complutense de Madrid (UCM), 28040 Madrid, Spain; 3 Hospital Infantil de Mexico Federico Gómez, Ciudad de Mexico 06720, Mexico; dr.homeromartinez@gmail.com; 4 Nutrition International, Ottawa, ON K2P 2K3, Canada

**Keywords:** salutogenesis, health assets, health promotion, participatory action research, rural health, community nursing, primary health care, community health

## Abstract

Salutogenesis focuses on factors that generate health and is a useful construct for identifying factors that promote health and for guiding activities to this end. This article describes health assets identified in a community diagnosis and how to leverage them with actions for improvement to deepen the understanding of this concept and its impact on health promotion. An intervention strategy was designed following the principles of participatory action research (PAR). The study was carried out in Mañaria (Basque Country, Spain) using semi-structured and in-depth interviews, participant observation, desk review, and photographs, alongside different participatory strategies. Twenty-six women were interviewed, 21 of whom were community inhabitants, and five were key informants who worked in public or private institutions. Participant recruitment stopped when data saturation was reached. Data were analysed through discourse analysis, progressive coding, and categorisation. Six meta-categories emerged, and for each of these categories, health assets were identified together with actions to improve the community’s health. The latter were presented by the community to the authorities to trigger specific actions towards improving the health of the community. Identification of health assets led to different actions to improve the health of the community including improving the existing physical and social environments, personal and group skills, and the promotion of physical, social, emotional and cultural well-being.

## 1. Introduction

Health promotion focuses on implementing policies that facilitate healthy choices and enable individuals and communities to have control over different activities that generate or promote health [1]. It involves reorientating health services and building environments and policies favourable to health as well as the development of personal skills and people’s participation so that they can exercise control over their own health [2,3].

Moreover, a community health diagnosis, which is intended to provide a detailed description of a community’s health, should be the basis for any intervention [4], and it can be a good starting point to detect community elements that promote health [5,6]. A community health diagnosis also helps to strengthen the links among different agents in the community (neighbours, service workers, health institutions, etc.) and represents an opportunity for a community to develop leadership and empowerment, facilitating action-oriented strategies [6,7].

This study was based on the concept of health as a universal right and a dynamic, subjective and individual experience of a person living in a community that shares economic, social and cultural characteristics and is affected by environmental factors and rules for coexistence. Health is understood as a relative state in which one is able to function effectively to express one’s greatest potential in the environment in which one lives [8] and involves the physical, social, emotional and cultural well-being of people in their own environments while contributing to the well-being of the whole community [9,10].

From a positive perspective, health has been reinforced thanks to the theory of salutogenesis (origin of health), one of the most solid foundations of health promotion [11,12]. Salutogenesis is a way of looking at health from that which generates it, and this facilitates individuals, families and communities to increase control over their own health and improve it. It can be very useful to guide efforts, to understand better how health originates and what and where the salutogenic factors are that accompany the person in her/his vital development, promoting capabilities and assets for health. The concept of a health asset is understood as a factor or resource that enhances the capacity of individuals or communities to maintain health and well-being [13]. Assets for public health are part of the heritage of a community and serve to maintain or improve health as the result of an organised effort of individuals, associations, organisations, institutions, and environmental or cultural resources [14].

The identification of opportunities to maintain or improve health is becoming a suggestive methodology for strengthening community action and community control over a community’s own health [15,16,17]. This is also what the World Health Organization (WHO) [1] proposes when it talks about making the healthiest options the easiest to choose. In this sense, the study presented here is based on a previous community health diagnosis [5,6] carried out to understand the health of the community of Mañaria from the women’s perspective. One of these specific objectives was to determine the situations that favour health in this community and to identify actions for improvement. Women’s perceptions were chosen because they can contribute to improving protection, promotion and self-care of both their health and that of the community in general through the identification of mechanisms that promote dialogue, agreement and negotiation between community members and institutions [18].

The research process, which was carried out following a participatory design, specifically, participatory action research (PAR) [19,20,21,22], involved three community actors: local administration, technical and professional resources (educational, social, health, economic, etc.), and community members [6,23,24,25]. All of them were in a privileged position to promote the health of the population due to the fact of their proximity to the community and accessibility to citizens.

Regarding local administrations, municipalities may influence and promote health through citizen planning and the creation of local policies that prioritise health and equity, thus assuming a central role in the establishment/promotion of healthy communities. This is because, in addition to managing services and developing regulations, they can promote the empowerment and participation of citizens [9].

Based on the community health diagnosis carried out by the same research team [6], the primary objective of this study was to identify the community’s health-promoting opportunities based on the strengths (i.e., health assets) identified by the women interviewed and to foster specific actions that would positively impact on the individuals’ and community’s health.

## 2. Materials and Methods

The community under study was Mañaria, located in the central–southeastern part of the Historical Territory of Vizcaya, Basque Country, in northern Spain. Throughout the study, the population of the community fluctuated between 507 and 522 inhabitants, distributed between the urban centre and the five neighbourhoods of the community.

The extension of the community is 17.73 km^2^, so the population density was 28.60 inhabitants/km^2^. Mañaria is located in a privileged environment, in a valley surrounded by mountains. Of the surface, 72.75% of it belongs to the Urkiola Natural Park within this municipality, witnessing how its beauty is threatened by the proliferation of mining operations that have already damaged it considerably.

Regarding the economy, the primary sector is represented by mining operations and small agricultural and livestock family farms. The service sector is very small. In relation to the epidemiological profile, chronic diseases related to the circulatory and cardiac systems, respiratory, musculoskeletal, digestive, urinary and dermatological systems stand out.

To achieve the proposed objective, the intervention strategy followed the principles of PAR. The intention was that the proposals identified in the initial community health diagnosis [6] would lead to actions for improvement and changes in the health and well-being of the community.

PAR is meant to promote change and strengthen people’s living conditions, including improvements in their environment [26], involving different social actors who, together with the research team, make it possible to construct significant knowledge in the scientific sphere, while at the same time developing the intervention and introducing changes for improvement in the situations analysed [27,28]. In this way, the people who participated in the study became “active subjects” of the research by involving them as protagonists in the process. PAR fosters a process of dialogue and effective communication between researchers and participants. Participatory health diagnosis is among the projects that are presented under the name of PAR in the socio-health field [28] as was the case in our study.

In the first phase, the research team was assembled, and the research proposal was developed. An initial report introduced the project to the local authorities and requested their collaboration. The second phase consisted of the elaboration of the health diagnosis, which had two phases: the first consisted of data collection, and after completing the data analysis, the main results were summarised.

Once the health diagnosis was completed, different participatory strategies were developed to involve the community. One strategy was respondent validation in which the researchers went back to the field to present and discuss the results obtained with the women participants and to identify the health assets, i.e., proposals for action that would lead to improvements in the negative aspects and reinforcement of the strengths identified. As part of this strategy, a period of further discussion with the informants was carried out as a way of validating the findings and to help refine or clarify any aspects of the researchers’ interpretation. This process enabled rectification and the gathering of new information in order to understand nuances. In addition, it enabled the inclusion of participants in the process, a feature implicit in PAR [26,28,29,30]. Subsequently, a final report was developed and written, incorporating the health problems, community strengths or health assets and women’s proposals for action.

A complementary strategy was the public presentation of the report to the rest of the community and local authorities. In addition to the researchers, the different actors involved in PAR were also part of this presentation (see Table 1). From that moment on, the third phase began in which different sectors in the community implemented actions to improve the community, linked to the proposed solutions put forward by the women who informed the health diagnosis. Among the relevant activities that were proposed and took place were addressing improvements to the natural environment and decreasing the noise in the quarry industry; promotion of community services including some health services; price control of the housing market (purchase/rent); maintenance and care of public spaces; local security; waste management; promotion of leisure and cultural activities that favour socialisation, etc.

The study sample included 21 female neighbours from the community identified by means of intentional sampling; in addition, five other women participated who were linked to networks of social and community associations involved in social cohesion and with the capacity to mobilise resources [6] (Table 1). The sampling technique sought to maximise the variability of the participants, thus contributing to a representative sample of the community [31], and stopped once saturation in the responses was reached [32].

Data collection methods included participatory observation, interviews (in-depth and semi-structured) and desk review, consulting multiple sources and complemented with photographs [6].

Participatory observations were recorded in a field diary [32] and were carried out at different times and places in the community (main square, ball court, neighbourhoods, cultural centre, etc.). The intention behind reaching out to these common spaces was to determine situations of collective coincidence (cultural, sports, festive activities, etc.) and to understand the context in which they took place. It should be noted that the main researcher lived in the community during the entire period of data collection and that she was fluent in the local language, Euskera.

The observation served to capture the social and cultural reality of Mañaria through the inclusion and interaction of the main researcher in the space and collective of Mañaria, the study of spontaneous speeches and behaviours during the time in which they occurred in order to obtain a broader knowledge of the situation and to discover the patterns of conduct and behaviour when being in direct contact with the Mañaritarras living in the same situations that the community under investigation lived. All of this facilitated the subsequent analysis and interpretation of the results.

In-depth interviews were recorded and transcribed prior to text analysis. Semi-structured interviews were also conducted as part of the ethnographic study. From these two sources, data related to health assets were extracted through a process of progressive coding and categorised into a series of broad concepts or meta-categories [6].

**Figure 1:**
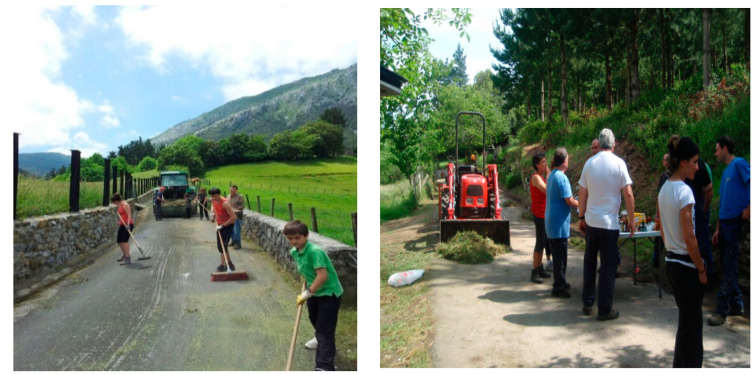
Neighbours in an auzolana (participatory community work). Participants or parents in the case of minors gave their consent to have their pictures taken.

The use of photographs was particularly useful, as these provided the opportunity to closely analyse moments of collective action as shown in Figure 1 [17,32,33,34,35].

The interviews and informal conversations were conducted in Spanish and Euskera, which enabled more fluid communication with the informants who usually speak these languages. Use of the local language also helped to build rapport and empathy, both of which are highly valued in qualitative interviews and participatory methodology [32].

The study was approved by the Ethics Committee of the Public University of the Basque Country-Euskal Herriko Unibertsitatea and observed the principles of the Declaration of Helsinki [36]. All interviews required oral informed consent from the participants, who were informed that anonymity and confidentiality would be preserved. Transcribed information was anonymised using letters/numbers for coding and analysis. Participants or parents in the case of minors gave their consent to have their pictures taken, to be used to illustrate aspects of the research, with no commercial purpose. To ensure the robustness of the research, the COREQ guidelines [37] and the criteria proposed by Calderón [38] were considered: validity, appropriateness, relevance and reflexivity.

## 3. Results

### 3.1. The Sample

The sample included 21 women with a mean age of 47 years distributed as follows: 20–24 years (*n* = 2), 25–54 years (*n* = 12), 55–69 years (*n* = 5) and >70 years (*n* = 2). All geographical areas of the community (inner city and five neighbourhoods) were represented in the sample. In addition to these 21 women, five additional key informants were selected, seeking to include professionals and social agents working directly for the community, promoting social interaction and health promotion among its members. Four of these were employees of the three main governmental institutions in the community: the city council, the social services and the health system. The fifth key informant was selected for her involvement in a civil association (i.e., Mañaria Bizirik). Twenty-three of the 26 women were permanent residents in the community; three did not live there but commuted daily and worked full-time in the community.

### 3.2. Health Assets

The following six meta-categories were extracted from the data analysis: population, from household to community economy, public and private spaces, habits and lifestyles, socialisation process and health care network [6]. In each of these meta-categories, strengths or health assets were identified as described below.

#### 3.2.1. Health Assets in the Meta-Category “Population”

The women described that new people had settled in the community, which included people who came from other localities. This was experienced in a positive way. The desire to live in a rural environment and the search for independent spaces with land were some of the reasons mentioned for choosing to live in this community. “We live a quiet life and Durango seemed bigger and we preferred something smaller. We liked it. At first it seemed too small…but it was the fact that it was a small town, like a village, that we liked” (I-2). The informants perceived that the birth rate and the presence of young people in the community had increased. “There has been a gap, years in which few children were born (in four years only one child has been born). Last year, however, eleven children were born” (I-1). “Now… young people are beginning to stay” (I-16).

Therefore, two health assets were identified:

- The recovery of the value of the rural environment due to the presence of two factors: (1) tranquillity, far from the stress of the cities, and (2) the search for independent housing, owning their own land or green areas;
- As a consequence of the above, an increase in the birth rate and changes in the demographic structure of the population were noted, leading towards a rejuvenation.

#### 3.2.2. Health Assets in the Meta-Category “from Household to Community Economy”

Regarding paid work, the general subjective feeling was a sense of satisfaction, because the informants liked what they do, because the tasks and functions they performed were more positive than those they carried out in the domestic environment and because the working conditions were more suited to their personal needs, thus allowing them to reconcile work and personal life. “… more than anything else, you disconnect from home, and you go home with a different feeling!” (I-5). Most of our informants perceived their economic situation as good or said that they were able to live well financially. By owning the flat/house in which they lived, having money saved or properties that provided support every month or help received in material goods (foodstuffs) from neighbours or relatives, this favoured the good economic situation of the family units. For some informants, living in a rural environment helped many people to benefit from economic support and assistance such as owning property, land, livestock or a vegetable garden. “Well, well… in general, well, I see it as good, because people, people in small towns already have resources: the vegetable garden, livestock or I don’t know what kind of rented flat” (I-15).

In Mañaria, work resulting from joint citizen participation, auzolana, was identified as an important health asset. The auzolana is deeply rooted in the culture of the community of Mañaria. An example was the work carried out by the Ermitauak (hermit in Spanish) group, which has been repairing the hermitages through auzolana since 1993. The maintenance of the hermitages is a cultural and tourist incentive, as it encourages visits from outsiders as well as being a personal and identity incentive, as they form part of the religious artistic heritage of the town. Moreover, the town council, considering that the auzolana can be a very rich tool for coexistence and neighbourly relations, has attempted to expand the existing experiences in Mañaria through an initiative launched in 2012 to promote and revitalise this practice, increasing its uptake with new proposals, which was still in place at the time of this study. This involves taking part in community tasks (cleaning neighbourhood paths, repairing damage, painting public spaces and railings in the centre of the community, cleaning windows in the ball court, etc.), which precisely serves the purpose of recovering old traditions, saving municipal expenses and enriching the atmosphere of the neighbourhood.

In summary, the following health assets were identified in this meta-category:

- Paid work if the following conditions were met:
  * The tasks performed are better valued than those carried out in the domestic sphere;
  * That it allows for family reconciliation;
  * That it guarantees private ownership (of housing and productive land or livestock);
  * That it is enough to allow for savings.
- Auzolana facilitates community coexistence, social cohesion and the establishment of effective collective commitments for the benefit of the community and public cost savings.

#### 3.2.3. Health Assets in the Meta-Category “Public and Private Space”

The opinion regarding the town centre was generally positive and the cleanliness, tidiness and aesthetics were rated satisfactorily. “Urbanistically, I don’t see anything missing. The main square has been restored and it is very nice. I love it. A few years ago, it was horrible, but now it is very nice. They are taking care to fix up some of the little houses…. There will be things to touch up, but I think it’s very nice. The only thing that was missing was a square, there weren’t any seats or anything…” (I-2). Regarding the neighbourhoods, the one most mentioned was Urkuleta, because it is a natural and peaceful space, suitable for walking, strolling or jogging. “… the Urkuleta area is good… I like it, we usually go for a walk…” (I-18).

As the current study evolved, improvement works were carried out on pavement drains, and zebra crossings were correctly located.

The city council carried out several public consultations on urban planning. One of them aimed to decide how access to the cemetery and the library should be improved, and another consultation aimed to decide the location of rubbish containers (in the town centre and in the neighbourhoods). In addition, to request improvements to the public transport line, the council proceeded to collect signatures from neighbours. Both the consultations and the collection of signatures were the steps prior to the materialisation of the changes and improvements.

Regarding safety, some informants felt that the level of security and trust was such that they did not even feel the need to lock their front door. “We live safe, half the day the doors are open…everybody comes in here…super safe!” (I-7). Many informants equated their sense of safety with tranquillity. “I live in peace and quiet in Mañaria, safe, fine” (I-17). Moreover, the fact that people know each other reinforced the feeling of safety; because there is trust, others would not hurt you, and it gave a feeling of protection: “It’s peaceful, it’s safer than in a big town, everyone knows you…” (I-5).

In this meta-category, the health assets identified were:

- Cleanliness, order and environmental aesthetics in community spaces;
- Safety in terms of preventing accidents in the streets with adequate urban access and security against intruders who may threaten private property. A sense of ease and trust in others arose as a consequence of this perceived safety;
- Citizen consultation by local managers and institutions prior to decisions affecting the community.

#### 3.2.4. Health Assets in the Meta-Category “Habits and Lifestyles”

In terms of food, Mañaria’s vegetable gardens, in addition to producing food locally, are a source of natural food for its inhabitants and a good way for future generations to learn about issues related to the origin of what is consumed. Many people in the community own a plot of land on which they grow agricultural products. According to participants, eating breakfast, lunch and dinner as a family can turn this family time into a stimulus that contributes to the promotion of proper food education by example and the development of social, emotional and interpersonal skills of its members. “It is taught at home, at the table, how to behave, how to eat, how to sit… it is difficult, but for us it is good and important” (I-16). Some informants explained the value of trying to carry out this act at home and in company. “I bring him (referring to her son) home from school to eat together” (I-2). The number of daily meals should not be less than three (breakfast, lunch and dinner), although new trends point to the need to increase this number to five. This is actually what we observed in our study: the aim was to encourage family sharing during meals, and the general adoption of five meals per day.

Regarding alcohol consumption, a repeated concept was that of “poteo and txikiteo”, equivalent terms rooted in the Basque Country that consisted of drinking small portions of wine (txikitos) or beer (zuritos) whilst standing with friends and every so often going to a different bar. In other words, moving along to several bars where one can have a glass of wine and enjoy some pintxos or tapas. In fact, in December 2013, an initiative was launched that tried to combine the habit of “potear” with singing, a tradition that is being lost, although not so long ago it was very common. The aim of the “poteo o txikiteo” is to recover old songs, learn new ones, and encourage interrelationships with other people and among the “cuadrillas” (groups of friends).

Regarding leisure time, fun, rest and personal development, these can be found in very different activities. The types of free time activities can be classified into two groups. The first type is of an individual, autonomous nature, based on listening, watching or interacting with media (television, listening to music, radio, etc.), which takes place in smaller, more intimate spaces such as the home. The second type is of a collective or social nature, which normally takes place in public spaces. The activities mostly carried out by women in their free time have to do with physical exercise, the development of imagination and memory, creativity, media monitoring and with a social nature. All of this will contribute to physical and psychological benefits, “I cycle every day to Urkiola. Without a bicycle I get itchy… if I don’t go to the mountains, I cycle. Walking too. I need to do something every day, like someone who smokes and needs to smoke” (I-6), and social benefits, “We go to Izurza every day (one hour), my niece and I” (I-1). The aim of Mañaria Town Council’s Department of Culture has been to offer cultural activities, considering through various consultations the interests and wishes of the population in order to adapt the offer to people’s needs.

In addition to the Town Council, leisure and free time activities are mainly organised by the Andra Mari Dance and Choir Association, the Santa Úrsula Association and the Pelota School as well as other groups, institutions and entities. Thus, extensive and varied activities are offered: occasional courses/workshops/lectures, annual courses, performances by the Kirikiño Choir of Mañaria, organ concerts, campaigns, sports, festivities, excursions/visits, performances, activities for children and young people, exhibitions, conferences, fairs and street markets, annual cultural week, etc. “There are less things but there are also parks, activities for children (learning English, etc.), in summer the udalekus, courses for older people…In this sense it (the village) is very good…, in a big village you don’t have it like that. There was also a kind of survey before, suggestions to find out what people wanted, so people asked for what they wanted…There are things: some handicrafts…” (I-5). For another informant, it was very positive that information about cultural activities was mailed out monthly. “I think that before there were not so many opportunities. Also, the information sheet we receive every month is very good, because you find out what’s going to happen. Before, they used to put up some posters in the street and…” (I-21).

The specific health assets identified in this Habits and Lifestyles meta-category were linked to:

- Natural and indigenous food; enjoying family meals at least three times a day;
- Moderate alcohol consumption linked to social activities that encouraged the revival of local traditions (i.e., singing);
- Cultural activities organised by local institutions;
- Sport and walks in contact with nature.

#### 3.2.5. Health Assets in the Meta-Category “Socialisation Process”

The socialisation process involves lifelong learning that takes place in various community settings, both formal and informal. All these scenarios coexist in Mañaria, and members of this community participate in them, alternatively or simultaneously.

For many years, the community of Mañaria had an educational facility, a rural school, and the experience of many of those who went to school or whose children did so was very positive. “Very much at ease!” (I-19). Mañaria offers a nursery service. The women are happy with this service, as it allows them to carry out other tasks (leisure, family or work), because it is considered a necessary service that every community should have, and also because it helps the children to get to know others in the same community, helps them to get to know their own environment and acquire security and facilitates family support when needed. Finally, the child-caregiver ratio was considered very appropriate. “Some mothers find it very convenient; I think it’s good, they have to work and it’s convenient for them to leave the children” (I-4). “As a village, this service has to be…” (I-17). “… having the children in the same community makes it easier for the family (grandparents, uncles, aunts…) to help out. The children know the environment, they adapt to their safe environment…” (I-17). “With small groups, parents must be delighted” (I-17).

In addition to the formal setting for people’s socialisation and sociability, there were other settings that are also involved in this process, closely linked to coexistence and integration. Many of the informants felt integrated into Mañaria’s community. The elements favouring this integration were related to personal qualities and abilities, to the fact of having a group of friends (the cuadrilla or “gang”) or children who have been the bridge for integration, in addition to the fact that people are open and they know how to help, collaborate and make others feel welcome. “We knew that we were the ones who had to open up…Wherever we go, we stay” (I-2). “It has been easy for us to feel at ease in the village, perhaps because of our child that you relate to mothers who also have other children of the same age as yours…I went out to the town square with our child, then other women started to appear, and we set-up a women’s group” (I-2). “I feel integrated. Yes, yes…in the end, you’re from outside, but I’m from Mañaria, from there too! And you see that people take you as if you were from here, that they care about you, that you are part of a community…” (I-14). Some informants valued very positively or positively the relationship they have with people in the community. “Relationships with people are very-very good. We know the people well, and we know who we can say something to and who we can’t say something to or who we can say things to in one way or another” (I-1).

Another element that was uncovered in this study dealt with the richness of coexistence and participation, focused on structured associationism. Many of the informants participated, in one way or another, in recreational and/or cultural activities organised in the community. “We have tried to participate in whatever has been organised in the community” (I-18).

The forms of participation in Mañaria ranged from mutual aid groups, associations that carry out leisure activities, training or provide services for other people to organised voluntary work. Many people took part in groups and associations in Mañaria: Andra Mari Dance Association and Choir, Espatadantza Dance Group, Santa Úrsula Retired People’s Association, Youth Assembly, Pelota Group and the Parents’ Association. Furthermore, in line with the enrichment of life in Mañaria, the celebration of different festive events established by the annual liturgical calendar were highlighted: the Three Wise Men, Santa Agueda, Txitxiburduntzi, Carnival, San Juan, San Martín, the Virgin, Santa Cruz, Santa Úrsula and Olentzero.

Three styles of encouraging participation were identified: (1) receiving suggestions, petitions and complaints; (2) citizen consultation; (3) bringing the town council closer to the base of the social fabric, giving everyone a chance and contributing a great deal to municipal management.

In Mañaria, the external settings for informal socialisation are usually the council premises, the old library, the Errose Bustintza Cultural Centre, the pensioners’ home, the ball court, the square and the church.

Therefore, in this meta-category “socialisation process”, the following health assets were identified:

- Formal settings, mainly the school and the nursery school;
- Informal settings: personal and institutional, linked to local traditions and ways of relating.

In both settings, socialisation was understood as a service to the community and as a way of learning and personal growth. Again, typical cultural elements of the area were noted (e.g., the “cuadrilla” as an informal group of stable relationships) that were highly engaging for those who participated in them.

#### 3.2.6. Health Assets in the Meta-Category “Care and Carers”

Regarding informal care, many of the women interviewed took care of the members living with them, because they were mothers and wives or partners and, therefore, had more say than the other members of the family unit in the management of health care. They were well aware of the care needs and situations in which the most vulnerable people often found themselves. This informal care arises in the family, the neighbourhood or close-by settings. Several informants referred to the way in which the elderly were cared for in the community of Mañaria. In general, the perception was that this population group in this community was well cared for, supported and accompanied by family and the neighbourhood network and in their own environment. The desire to be cared for by relatives was closely linked to the desire to stay at home. Several informants highlighted the farmhouse or the house as the family nucleus and the meeting place for family members. “I think that, at the village level, the elderly are very well looked after in their homes, among the members of the household, taking turns” (I-1). “In Durango, just like here, there are many elderly people, but here they are less lonely, this concept of family, respect, not leaving them alone… In Durango there are a lot of lonely people. It is very, very sad. It’s a value here, I think people are healthier and more accompanied…less lonely. It’s the same concept that existed before in the family…. respect, affection… it has always caught my attention” (I-7). In rural contexts, such as Mañaria, networks of neighbours or friends acquire great importance for informal care. “…or because they had a neighbourhood network that supported these people in order to take them for tests or to get medication at the surgery or to take them to the doctor in Durango…” (I-23).

Moreover, Mañaria has a consultation room, made up of a team of two professionals: a doctor and a nurse. There was great satisfaction with the work of this team, including the fact that they had been working for a long time in this community with continuity of care, thus favouring a better knowledge of the people in the community and vice versa. “…They (health care professionals) have been there for 18 years, and I was very comfortable with them” (I-1). “There was one who was there previously for a lot of years, yes, and everything was very good. In the end after so many years, you get to know them” (I-21). Home care and the frequency of home visits by the clinic team was a positively valued task. “When my mother was widowed, she came to live here with us and spent 10 years at home and they came here to look after her. They came almost every day, both the doctor and the nurse: wonderful!” (I-10). Some women in our study, in their role as informal caregivers, have felt alliances with the practice team by receiving training in caregiving from the professionals. “They taught me how to administer morphine” (I-10). “They taught me how to do my mother’s dressings” (I-11). Many of the women interviewed, when giving their opinions about the professionals at the clinic, mentioned the attitude and the kind and empathetic treatment they received from them. “…. with a lot of affection and fine (I-11). “… happy, they responded well. When my father was ill, they were concerned” (I-17). A basic element inherent to good communication is language; in the context we analysed, it was knowledge of the Basque language. Some informants addressed this issue and found it positive that health professionals knew how to communicate in their language. “The new one is Basque…I am comfortable with the new one” (I-1). “The doctor now knows Basque and it’s better. The nurse does too. And I see that as positive” (I-9).

In relation to this meta-category, the following health assets emerged:

- Being in their usual dwelling and remaining in it, favoured care, in general, of the family nucleus, especially care of the elderly, and it made it easier for women carers to perform this function without interruption;
- The provision of continuous and prolonged health care by the same professionals. This encouraged treatment as well as personalised care, which led to a therapeutic bond of trust. Another element that favoured a relationship of trust was the professionals’ knowledge of the local language.

In addition to the assets identified in each of the meta-categories, this study describes the actions carried out in the community by the different sectors of the community, and it was observed that these actions were actual health assets, because they were aimed at the individual and collective well-being of this community. Table 2 shows these improvement actions and their correlation with health assets and health promotion.

## 4. Discussion

The present study was part of a broader investigation [5,6] that has described and analysed the health of the community of Mañaria from the perspective of its women, identifying health problems, needs and strengths of this community as well as proposals to improve the health of the community. In particular, this paper focused on the strengths identified, i.e., health assets. The use of the term “health assets” implies a change in the way of thinking and looking at the contexts in which the members of a community live. Health assets were identified based on a community health diagnosis as the potentialities and capacities for health or those components that produce health [14] by valuing the talents, resources or assets of the people and of this community. Health assets were identified based on the analysis of meta-categories that emerged from the interviews and the participatory observation process based on the salutogenic approach [11,12]. In this study, the actions carried out by different community sectors were collected and multiple dimensions were analysed in line with the approaches to the social determinants of health (SDH) as defined by Dahlgren and Whitehead [39]. Health assets identified in this rural setting and the actions carried out from different sectors that connect and energise these assets provide some keys to addressing health promotion at the community level [15] and have served to understand the multiple factors (population, economic, social, cultural, habits, etc.) that can be related to health, generating well-being, and healthy development in the community.

In our study, health assets were classified according to the emerging meta-categories in the health diagnosis, and it is worth highlighting that they were aligned with the main sources of assets proposed by McKnight et al. [13,40], which include people, associations, institutions, services, cultural expressions, spaces or places and elements of the local economy.

The present study was based on the assumption that health is not the exclusive competence of health professionals but is influenced by living conditions, the environment and individual and collective behaviours. There is a growing body of evidence on how these different determinants affect both life expectancy and the probability of becoming ill [14]. In our case, elements such as urban or domestic factors appeared to be strongly linked to the concept of global well-being and, therefore, to a broad conceptualisation of health.

For this rural community, a relevant asset was that people were moving to live there, because this was a determinant for the growth and rejuvenation of the population, thus impacting on the demographic structure. In addition, the people who were part of the community were also considered as assets: community agents, family members, friends, neighbours, associations, certain professionals or the local government. These were considered to be people who bring together and unite different sectors of the community, who seek individual and collective well-being [13,41,42].

This study clearly connected several elements of the local economy with the generation of health [13,43,44]. The spaces or places in Mañaria where daily activities were carried out were also described, and the informants considered them to be determining factors in health, as they represent places of coexistence and daily life [45,46]. However, the community at large was also considered to be safe, and this promoted the physical and material integrity of people, which are fundamental requirements for them to be able to expand their capacities and effectively exercise their freedoms [47]. Likewise, assets were identified in the resources related to habits and lifestyles (food, physical activity, leisure, etc.) [42].

Another of the study’s contributions is related to the link between health and socialisation and, within this, coexistence, understood as “the interpersonal and social dynamic, based on dialogue, trust and solidarity, which allows all people to feel part of a society and to enjoy their human rights” [13]. In this sense, everything that favours personal relationships is considered an asset for health. An example of this was the auzolana, the aim of which is to improve relations between neighbours and to cover by one’s own personal work whatever the municipality cannot provide or assume. Other research has also addressed the effectiveness of community activities in health promotion [48,49].

One dimension that was positively valued by the participants was related to health care services. The assets of the Mañaria clinic and the importance of the informal care network, which included family, relatives, neighbours and friends, were identified and highly valued.

Regarding the actions implemented (Table 2), these were aimed at making the community more attractive, comfortable, lively, fun, rich, accessible and cultured, as interventions have been carried out to promote services and improvements to the landscape, participation in community budgets, sustainable mobility, physical exercise and spaces adapted to this and to foster socialisation of people.

Furthermore, the role that some community actors have in addressing community health issues not only from an individual but also from a collective vision was highlighted in this study. For example, it was clear that the objective of the local government (i.e., town council) was not only to cover demands but also to have a proactive vision—with an explicit commitment to understanding the needs and wealth of the locality it manages at the community level in order to establish collaborative work processes with other community agents with the aim of generating health and well-being in the community of Mañaria. In line with previous authors [50], some of the community processes identified in Mañaria and the improvement actions implemented (Table 2) did not only consist of agreeing and coordinating the different projects, programmes or actions developed in the community. Thus, bonds were generated together with relational processes, strengthening the cohesion of the people and groups that live and work in the community. Sometimes these were structured actions and at other times they are more “informal” actions; however, they were fundamental for the community development of any community. As Carmona and Rebollo [51] point out, community action is “the dynamisation of cooperative social relations between the members of a given area or space of coexistence in order to improve people’s daily well-being”.

A fact that stands out in our research was the importance and influence of local culture in the configuration of health assets. Several of the traditions and customs observed, for example, the auzolana, are in themselves health assets, as they promote community cohesion and participation to achieve better standards of well-being and health.

Recent publications have reported similar experiences to those identified in our study related to health assets [14,52]; however, in contrast to our study, these studies were not contextualised in rural areas. Thus, this context provides an original data set to complement proposals by previous studies.

In short, and in line with other authors [53], the assets and actions identified in Mañaria help to make this community a healthier place. In addition, this study has enabled us to understand and build integral knowledge of this environment, placing value on its assets and resources, so that healthier lifestyles can be facilitated for everyone.

To identify health assets, there are information gathering techniques that are more appropriate than others [17]. In our PAR, we resorted to qualitative methods including interviews, participant observation, analysis of documentation (desk review) and photographs. These techniques allowed us to identify health assets, highlighting those elements that generated well-being at the individual, group or community levels [34,35]. Strategies to enhance the participation of all actors involved and the horizontality of the research process are also elements of social cohesion and generators of participatory behaviours.

As the study suggests that the community of Mañaria promotes the development of the capacities of its population, from an intersectoral perspective, future lines of research and intervention strategies could focus on policies oriented towards health promotion developed in an intentional [9], planned and participatory way. It would be worthwhile to convey the health assets approach to the local government sphere and to promote intersectoral meetings to jointly seek common ground to continue promoting health in Mañaria. In addition, knowledge of a community’s assets or strengths can be useful in the therapeutic process of the Mañaria clinic (“health assets recommendation”) [54]. Recommendations related to health assets can be made from the nursing and medical practice [54,55]. In this manner, the changes that are necessary for people to improve their health can be facilitated. Finally, it would be interesting to develop a map of health assets, constructed in a participatory fashion to further advance goals related to health promotion and well-being [56].

As limitations of the study, it should be highlighted that the identification of health assets corresponds to a specific chronological moment, that is, they are synchronic, and could vary over time. It would be interesting to document if the health assets vary over time in response to the changing social reality of the community, that is, to carry out a diachronic validation. Moreover, it would be interesting to include men’s voices. Undoubtedly, their participation could provide elements of contrast and similarities that would enrich the literature around health assets.

## 5. Conclusions

Based on the findings of this study, we can conclude that a participatory community health diagnosis was a useful tool for identifying health assets and promoting actions for improvement aimed at responding to the needs identified by the population and improvement of the health of the community. With all the assets identified and the implementation of actions for improvement, Mañaria showed its contribution to the promotion of:

- Policies and activities that made it easier for those in the community to have control over what generates health;
- Physical and social environments favourable to health, expanding community resources that enable and empower people to support each other in day-to-day functions, allowing them to develop their maximum potential;
- Personal skills and participation of people, so that they could manage their own health;
- The physical, social, emotional and cultural well-being of the people of Mañaria in the community environment, while contributing to the well-being of the whole community.

## Figures and Tables

**Table 1:** Actors involved in PAR.

Description	Number
Women participants	21
Professionals in social and health sectors	3
Members in the association “Mañaria Bizirik”	1
Council: Councillor of Culture	1
Researchers	3

**Table 2:** Assets and actions implemented in the community grouped into meta-categories and their impact on health promotion.

Metacategories	Health Assets	Actions Implemented [6]	Community Health Promotion
The Population	- Re-evaluation of the rural environment - Demographic changes towards the rejuvenation of the population	- Urbanisation improvement - Opening of a science museum	The promotion of services and landscape improvements can be favourable for people who choose to live in small communities, such as in this study’s context, or for people living in this community to feel more at ease.
From Household to Community Economy	- Paid work - Auzolana	- Opening of a hotel restaurant - Auzolana sessions. - Participatory budgets	The promotion of services, the possibility of the auzolana and the invitation to participate in budgets to help the economy of the community and make people feel linked to it and involved in it.
Public and Private spaces	- Cleaning - Security - Citizen consultations	- Expansion of public transportation	The promotion of public transport services encourages sustainable mobility and contributes to improving environmental health.
Habits and Lifestyles	- Natural and autochthonous food - Poteo and singing - Cultural activities - Exercise and sports in contact with nature	- Gymnastics offer - New sports facilities	The possibility of physical exercise activities and spaces adapted to it promotion of physical, mental and social well-being.
Socialisation Process	- Promotion of socialisation in formal spaces: school and nursery school - Promotion of socialisation in non-formal spaces: square, library, pelota court, etc.	- Organisation of leisure and free time activities for children - Offer of a cultural place for adolescents in the community - Offer of training sessions aimed at the entire community	The parents’ group organises activities that promote the health of their children and the integration of people into the community. It also promotes healthy socialisation. Adolescents have a space that is appropriate for them based on a participatory initiative. They have been promoters of their own health.
Health Care Resources	- Permanence in their usual dwelling, especially for elderly people, and care by relatives - Continuity of care over time by health professionals	- Paediatric emergency service request - Offer of health education sessions	Promoting paediatric services. The offer of health education sessions is one health promotion tool that empowers people to take control of their own health.

## Data Availability

The data presented in this study are available upon reasonable request from the corresponding author. The data are not publicly available in deference to participants, who were not informed that their replies, even when de-identified, would be made publicly available.
